# Photodithazine-Mediated Antimicrobial Photodynamic Therapy: A Systematic Review of Efficacy and Applications

**DOI:** 10.3390/ijms26168049

**Published:** 2025-08-20

**Authors:** Rafał Wiench, Jakub Fiegler-Rudol, Kinga Grzech-Leśniak, Dariusz Skaba, Josep Arnabat-Dominguez

**Affiliations:** 1 Department of Periodontal Diseases and Oral Mucosa Diseases, Faculty of Medical Sciences in Zabrze, Medical University of Silesia, 40-055 Katowice, Poland; rwiench@sum.edu.pl (R.W.); s88998@sum.edu.pl (J.F.-R.); 2 Laser Laboratory, Department of Integrated Dentistry, Faculty of Dentistry, Wroclaw Medical University, 50-425 Wroclaw, Poland; 3 Department of Periodontics, School of Dentistry, Virginia Commonwealth University VCU, Richmond, VA 23284, USA; 4 Faculty of Medicine and Health Sciences, Dental School, University of Barcelona, Investigator of the IDIBELL Institute, 08908 Barcelona, Spain; joseparnabat@ub.edu

**Keywords:** antimicrobial photodynamic therapy, photodithazine, photodiathazine, antimicrobial resistance, biofilm, *Candida albicans*, *Staphylococcus aureus*, denture stomatitis

## Abstract

Antimicrobial resistance is a critical global health issue exacerbated by biofilm-associated infections that often resist conventional therapies. Photodithazine-mediated antimicrobial photodynamic therapy (PDZ-aPDT) has emerged as a promising alternative, demonstrating a broad-spectrum antimicrobial efficacy against multidrug-resistant bacteria and fungi, including those in biofilms. This systematic review evaluates the efficacy, safety, and clinical applications of PDZ-aPDT by synthesizing evidence from preclinical and clinical studies. Databases including PubMed, Embase, Scopus, and Cochrane were systematically searched, resulting in the inclusion of 13 studies for qualitative analysis. PDZ-aPDT consistently reduced the microbial burden in various models, including oral candidiasis, denture stomatitis, acne, and infections related to medical devices. Synergistic combinations with conventional antimicrobials and adjunctive therapies (e.g., DNase I) further enhanced its effectiveness. However, the evidence base remains limited by methodological variability, small sample sizes, and short follow-up periods. Future research should focus on rigorous clinical trials with standardized protocols and extended follow-up to establish definitive efficacy and safety profiles, facilitating a broader clinical implementation in combating antimicrobial resistance.

## 1. Introduction

**Figure 1:**
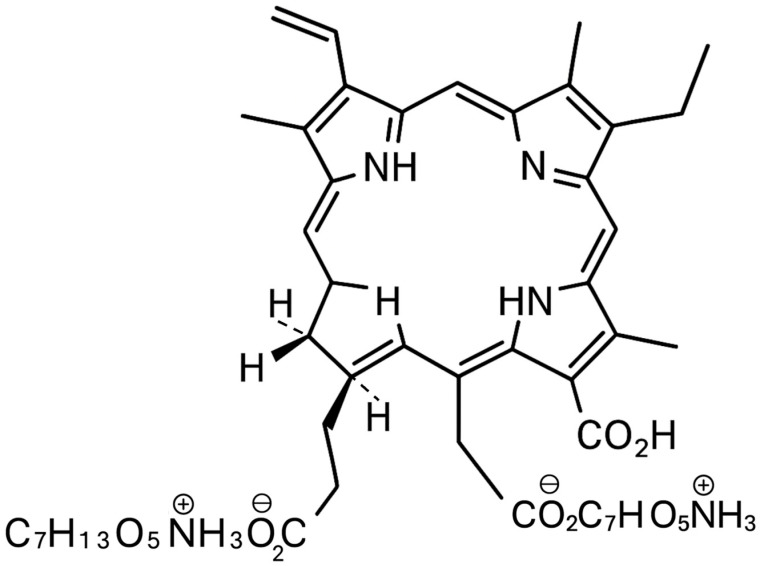
Structure of PDZ (image created by the author using Microsoft Office Tools, Microsoft Office 365).

The rapid rise in antimicrobial resistance is recognized as one of the foremost global public health threats of the 21st century [1,2]. Conventional antibiotics and antifungal agents are increasingly compromised by the emergence and spread of multidrug-resistant bacteria and fungi, making the treatment of common infections ever more challenging and costly [3,4]. In addition to planktonic (free-floating) forms, many clinically relevant microorganisms persist within complex biofilms, structured communities encased in an extracellular matrix, that are inherently tolerant to traditional therapies and are a major cause of chronic and recurrent infections in the oral cavity, skin, and medical devices [5,6,7]. In the search for novel, effective alternatives to conventional antimicrobials, antimicrobial photodynamic therapy (aPDT) has emerged as a promising non-antibiotic strategy. aPDT relies on the administration of a photosensitizing agent followed by illumination with light of a specific wavelength, which in the presence of oxygen leads to the generation of highly reactive singlet oxygen and other reactive oxygen species (ROS) [8,9,10]. These ROSs induce oxidative damage to microbial cell structures, ultimately resulting in cell death. Importantly, aPDT has a multi-targeted mechanism of action and does not promote the development of microbial resistance, making it attractive for the management of resistant and biofilm-associated infections [11,12]. Among the various photosensitizers explored for antimicrobial applications, Photodithazine (PDZ), a water-soluble chlorin e6 derivative, has garnered increasing attention for its potent photodynamic activity, favorable safety profile, and versatility across a range of clinical settings [13,14]. Figure 1 shows the structure of PDZ. It is a bis-N-methylglucamine salt of chlorin e6. PDZ is a mixture of di-N-methyl-D-glucosamine complexes, mainly containing Chl e6 (60%) along with Chl p6 and purpurins 7 and 18 [14].

PDZ is characterized by a high quantum yield of singlet oxygen upon red light activation (typically at 660 nm), good tissue penetration, and minimal cytotoxicity in the absence of light (dark toxicity). Its demonstrated antimicrobial spectrum is broad, encompassing Gram-positive and Gram-negative bacteria, drug-resistant pathogens such as methicillin-resistant *Staphylococcus aureus* (MRSA), and fungal species including *Candida albicans*, both in planktonic and biofilm states [15,16,17,18,19,20,21]. Recent experimental and clinical studies have explored diverse applications of PDZ-aPDT, including the management of oral candidiasis, denture stomatitis, periodontitis, cutaneous infections such as acne, and device-related biofilms [17,18,19,20,21]. Innovative protocols combining PDZ-aPDT with adjuvant therapies, such as enzymes (DNase I), antifungals (fluconazole, nystatin), or antibiotics (metronidazole), have further expanded its potential, showing synergistic effects and an enhanced disruption of biofilms and extracellular polymeric matrices [17,18,19,20,21]. Moreover, PDZ’s clinical formulations (solutions and gels) allow for convenient topical application, increasing its translational potential [18,19,20]. Despite these advances, the available evidence regarding the efficacy, safety, and optimal use parameters for PDZ-aPDT remains fragmented across a variety of preclinical and clinical models. No comprehensive synthesis has yet to critically examine the range of experimental conditions, microbial targets, and clinical indications in which PDZ-aPDT has been evaluated, nor has the quality of existing studies been systematically assessed [17,18,19,20,21,22]. Therefore, the primary aim of this systematic review is to comprehensively evaluate the efficacy and therapeutic applications of PDZ-aPDT across preclinical and clinical studies. This review summarizes the infectious models in which PDZ-aPDT has been tested, compares its antimicrobial effectiveness to standard therapies and alternative photosensitizers, and analyzes key parameters influencing outcomes such as photosensitizer concentration, light source specifications, and treatment protocols. Additionally, it assesses the safety profile, host tissue response, and risk of resistance, while identifying the potential of combinatorial and adjunctive strategies to improve clinical efficacy. By highlighting methodological strengths and limitations in the current literature, this review aims to clarify the role of PDZ as a photosensitizer, provide recommendations for optimal application, and support further research and clinical translation in the context of rising antimicrobial resistance.

## 2. Materials and Methods

### 2.1. Focused Question

The PICO framework [23] was used to guide the research question: Among individuals affected by microbial infections (Population), can an aPDT utilizing PDZ as a photosensitizer (Intervention), when compared with standard antimicrobial therapies, other photosensitizers, or the absence of aPDT (Comparison), result in more favorable outcomes such as reduced microbial burden, increased antimicrobial effectiveness, or enhanced clinical recovery (Outcome)?

### 2.2. Search Strategy

This review, registered with PROSPERO (ID: CRD420251085052) [24], was conducted in accordance with the PRISMA 2020 [25] guidelines to maintain high standards of transparency and methodological quality. A systematic search was carried out across multiple electronic databases, PubMed/Medline, Embase, Scopus, and the Cochrane Library, to identify the relevant literature on the use of PDZ-aPDT for managing microbial infections. The comprehensive search strategy is illustrated in Figure 1. To capture all pertinent studies, a team of three reviewers independently applied a predefined set of search terms and MeSH descriptors focused on Photodithazine and antimicrobial photodynamic interventions. Only articles published in English were considered, regardless of the year of publication. The selection process involved a two-step screening: an initial title and abstract review followed by a full-text assessment, conducted independently by two reviewers using established inclusion and exclusion criteria (summarized in Table 1). Additionally, references of the included articles were manually screened to identify any further eligible studies not captured in the database search.

### 2.3. Study Selection Process

To ensure methodological rigor and minimize the risk of bias, all retrieved studies were independently evaluated through a structured, multi-phase screening process by three authors. The initial screening of titles and abstracts was conducted using predefined inclusion criteria tailored to the review’s objectives. Discrepancies or conflicts in study selection were resolved through discussion and consensus to promote consistency and reliability in the decision-making process. The inclusion criteria were specifically designed to identify scientifically robust studies that investigated the antimicrobial efficacy of PDZ-aPDT. This review included experimental studies, either in vitro or in vivo, that examined the antimicrobial or biofilm-disruptive effects of PDZ as the primary photosensitizer within aPDT protocols. Eligible studies assessed outcomes such as bacterial or fungal load reduction, microbial viability, or the disruption of pathogenic biofilms. Studies exploring synergistic interactions between PDZ-aPDT and conventional antimicrobial agents, as well as those employing control groups (e.g., no treatment, placebo, or alternative photosensitizers), were also considered. Only studies that clearly reported the target microorganisms, described the treatment parameters, and included post-treatment evaluations of antimicrobial efficacy or biofilm response were included. Excluded from the review were non-peer-reviewed materials such as conference abstracts, case reports, editorials, opinion pieces, book chapters, and unpublished theses. Studies lacking scientific rigor, not written in English, or lacking relevant control or comparison groups were also excluded. In addition, research that did not use PDZ as the photosensitizer, studies evaluating unrelated technologies or therapies, and experiments involving non-pathogenic organisms or models lacking clinical relevance were not considered.

### 2.4. Assessment of Risk of Bias in Included Studies

To ensure a transparent and unbiased selection process, the screening of titles and abstracts identified during the database search was independently carried out by three reviewers. Inter-reviewer reliability was quantified using Cohen’s kappa coefficient to objectively measure the consistency of study inclusion decisions [26]. Discrepancies between reviewers were resolved through structured discussions aimed at reaching a consensus. This systematic approach was implemented to minimize the risk of selection bias, strengthen methodological integrity, and improve the overall reliability of the study identification process. The use of multiple independent reviewers and statistical agreement measures enhanced the rigor of this review on PDZ-aPDT and its therapeutic applications.

### 2.5. Quality Assessment

The methodological rigor of each included study was independently assessed by three reviewers using a structured quality appraisal framework. This evaluation was based on a predefined checklist consisting of nine critical domains, as outlined in Table 2. For each criterion met, a score of 1 was assigned; unmet criteria were scored as 0. This binary scoring approach produced a cumulative quality score for each study, with total scores ranging from 0 to 9. Based on these totals, studies were categorized as having a high (0–3), moderate (4–6), or low (7–9) risk of bias. Any discrepancies in scoring among reviewers were discussed collaboratively, with input from a fourth reviewer when a consensus could not be reached. The assessment process was guided by principles from the Cochrane Handbook for Systematic Reviews of Interventions [27]. Results of the risk-of-bias analysis are summarized in Table 2. No studies were excluded solely due to a high risk of bias. Of the nine studies included, seven were classified as low risk. Studies rated as moderate risk typically lacked adequate detail regarding control groups, statistical methods, or the transparent reporting of outcomes and funding disclosures. Evaluation criteria included the following: (1) clear documentation of the origin, purity, or synthesis method of PDZ used in the study; (2) description of the administration protocol, including concentration of PDZ, method of application, and incubation or exposure duration; (3) detailed reporting of the light source used in aPDT, including key parameters such as wavelength, energy dose (fluence), and exposure time; (4) evidence of microbial uptake or interaction with the photosensitizer prior to light activation, where applicable; (5) comprehensive description of experimental conditions, including microbial strain/species, culture methods, inoculation models, application site and environmental parameters; (6) inclusion of relevant control groups, such as untreated controls, light only, photosensitizer only, or comparator antimicrobials; (7) use of appropriate and clearly described statistical methods for analyzing antimicrobial efficacy or biofilm disruption outcomes; (8) transparent reporting of all measured outcomes, with no evidence of selective outcome reporting or unexplained missing data; and (9) disclosure of funding sources and any potential conflicts of interest that could influence the study design or interpretation.

### 2.6. Data Extraction

Following consensus on the final set of studies for inclusion, three reviewers independently conducted data extraction using a predefined, standardized protocol to maintain accuracy and consistency. For each selected study, critical information was collected, including the first author, year of publication, study design, characteristics of the experimental model or microbial species, and descriptions of both experimental and control conditions. Where applicable, data on follow-up periods and key therapeutic outcomes, both primary and secondary, were compiled. Particular attention was given to the technical parameters of PDZ-aPDT, including the concentration of the photosensitizer, detailed specifications of the light source (e.g., wavelength, power density, and total energy dose), mode of administration, and any adjunctive interventions (e.g., antibiotics). Procedural aspects such as irradiation duration, application method, and frequency of treatment were also thoroughly documented to enable meaningful cross-study comparisons.

### 2.7. Study Selection

**Figure 2:**
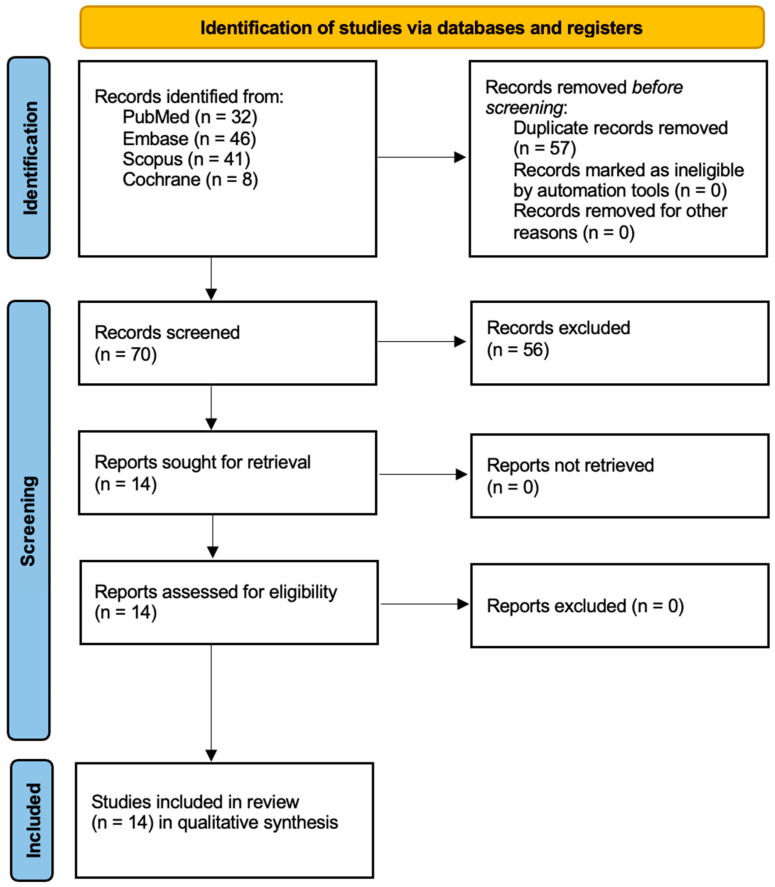
Prisma 2020 flow diagram.

In Figure 2, the PRISMA flow diagram illustrates the process of the study selection for a systematic review. Initially, 127 records were identified through four databases (PubMed, Embase, Scopus, and Cochrane Library). After removing 57 duplicate records, 70 records remained for screening. Of these, 56 were excluded based on their titles or abstracts, leaving 14 reports to be retrieved and assessed for eligibility. All 14 reports were retrieved, and upon assessment, one report was excluded because it was a case report [41]. Ultimately, 13 studies were included in the qualitative synthesis for the review.

## 3. Results

### 3.1. Data Presentation

Table 3, Table 4, Table 5 and Table 6 offer a detailed and organized presentation of the results from the nine selected studies, encompassing an overall summary, important clinical outcomes, and essential methodological information, including study designs and descriptions of treatment groups.

### 3.2. Overview of Study Characteristics

Table 3 presents an overview of the included studies.

### 3.3. Main Study Outcomes

Table 4 presents the main outcomes of the included studies.

### 3.4. Characteristics of Light Sources and Photosensitizer Used in aPDT

Table 5 summarizes the essential methodological aspects of aPDT as applied in the selected research, including a breakdown of light source specifications (wavelength, energy fluence) and detailed information on the use of PDZ as the photosensitizer, such as its administered concentration, delivery method, and pre-irradiation incubation period. Table 6 offers an in-depth overview of the chemical and functional attributes of PDZ relevant to its application as a photosensitizer in aPDT protocols.

## 4. Discussion

### 4.1. Results in the Context of Other Evidence

Photodithazine^®^ is a second-generation photosensitizer comprising an N-dimethylglucamine salt derivative of chlorin e6, which improves water solubility, enhances cellular penetration, and prevents aggregation at concentrations up to 120 mg/L compared to first-generation photosensitizers [30,42,43,44,45]. Optimally absorbing at 660 nm, it efficiently penetrates deeper tissues and, when activated by red light, generates ROS through both Type I and Type II photodynamic mechanisms, causing oxidative damage to microbial cellular structures such as membranes, proteins, and nucleic acids [18,45,46,47,48,49,50]. Photodithazine^®^ rapidly clears from the body, reducing skin photosensitivity effects compared to conventional photosensitizers like hematoporphyrin derivatives and localizes predominantly in the endoplasmic reticulum and Golgi apparatus, disrupting critical cellular processes [42,43,47,48]. Its clinical effectiveness is strongly supported by randomized trials demonstrating superior microbiological efficacy in treating oral candidiasis, notably denture stomatitis, where PDZ-aPDT achieved bacterial load reductions of 1.98 log_10_ on the palate and 1.91 log_10_ on dentures, substantially surpassing conventional nystatin treatment [30,49,50].

Similarly, preclinical murine studies confirmed its superior performance, achieving complete remission of oral candidiasis lesions and a 3 log_10_ reduction in *Candida albicans* viability [35,36,51,52]. Recent studies have also revealed potential in acne treatment, with Photodithazine^®^ combined with micro-LED technology effectively reducing acne lesions and key inflammatory biomarkers such as interleukin-1α, IL-1β, tumor necrosis factor-α, and IL-8 [16]. Moreover, Photodithazine^®^ exhibits broad-spectrum antimicrobial activity, including efficacy against methicillin-resistant and -sensitive *Staphylococcus aureus* strains at low concentrations (75–100 mg/L), and importantly, it demonstrates effectiveness against fluconazole-resistant *Candida albicans* strains. In biofilm-associated infections, it effectively targets multispecies biofilms comprising *Candida albicans*, *Candida glabrata*, and *Streptococcus mutans*, achieving substantial reductions in colony viability, with an enhanced efficacy observed when combined with DNase I enzyme, resulting in a 4.26 log_10_ reduction for susceptible strains and a 2.89 log_10_ reduction for resistant strains [30,31,32,33,34,35]. Preclinical safety evaluations in porcine models have confirmed minimal toxicity, showing only mild inflammation without systemic adverse effects, rapid clearance, and negligible renal accumulation [51,52].

Comparative studies with conventional antimicrobials like nystatin have shown equivalent clinical outcomes but superior microbiological efficacy, although recurrence of infection indicates the necessity of maintenance or combination therapies. Despite promising therapeutic potential, Photodithazine^®^ faces limitations including inadequate deep tissue light penetration, reduced efficacy under hypoxic conditions, and requirements for specialized equipment and trained personnel, potentially limiting broader clinical implementation, particularly in resource-constrained settings [53,54].

Regulatory approval for Photodithazine^®^ has been obtained in Russia for various clinical applications including malignant tumors and antimicrobial therapy, though limited international acceptance highlights the need for standardized protocols and larger clinical trials [54,55,56]. Ongoing research continues to explore promising future directions, including combination therapies with DNase enzymes, antimicrobial peptides, conventional antibiotics, and advanced nanotechnology-based delivery systems to enhance targeting precision and minimize systemic exposure, as well as sophisticated micro-LED and fiber-optic technologies for precise and improved illumination [57,58,59,60,61,62,63]. Further exploration of fractionated light delivery and combined modalities like pulsed electric fields or ultrasound may overcome current limitations, expanding Photodithazine^®^’s therapeutic reach. Thus, Photodithazine^®^ emerges as a valuable alternative or adjunctive approach to conventional antimicrobials, addressing global antimicrobial resistance challenges through its favorable safety profile, multi-target ROS-mediated mechanism, broad pathogen spectrum, and continued technological advancements in therapeutic application [40,41,42,43,44,45,46,47,48,49,50,51,52,53,54,55,56,57,58,59,60,61,62,63,64].

### 4.2. Limitations of the Evidence

Despite encouraging results, the body of evidence supporting PDZ-aPDT is constrained by several notable limitations. First, significant heterogeneity exists among the included studies, particularly regarding experimental models (in vitro, animal, or clinical), photosensitizer concentrations, irradiation protocols, and outcome measures, making direct comparison and synthesis challenging. Most of the available research is preclinical, with only a few well-designed clinical trials, which restricts the ability to generalize findings to broader patient populations. Short follow-up periods and small sample sizes further limit insight into the long-term safety and sustained efficacy of PDZ-aPDT. Additionally, inconsistencies in the reporting of adverse events and variable definitions of clinical endpoints may result in the underestimation of potential risks or overestimation of therapeutic benefits. The lack of standardized control groups and limited direct comparisons with established antimicrobial therapies or alternative photosensitizers create further uncertainty regarding the relative efficacy of PDZ. Collectively, these limitations underscore the need for future large-scale, rigorously designed clinical trials with standardized protocols and long-term follow-ups to robustly determine the clinical value and safety profile of PDZ-aPDT.

### 4.3. Limitations of the Review Process

Although this systematic review was conducted according to PRISMA 2020 guidelines and incorporated rigorous methodological safeguards, several limitations of the review process should be acknowledged. Restricting the search to English-language publications may have introduced language bias and led to the exclusion of relevant studies published in other languages. The omission of gray literature, such as conference proceedings, dissertations, and unpublished data, may also have contributed to publication bias, potentially skewing results toward studies with positive or significant findings. Additionally, the relatively small number of eligible studies, many of which were preclinical, limits the ability to draw robust conclusions applicable to clinical practice. The marked heterogeneity among study designs, intervention protocols, and outcome measures further precluded the possibility of conducting a quantitative meta-analysis. While multiple independent reviewers participated in screening and data extraction to reduce selection bias, the use of subjective quality assessment tools introduces the potential for reviewer bias. Lastly, the variability in reporting and lack of long-term data in many included studies limits the overall strength and generalizability of the review’s findings. A major limitation of this systematic review is the heavy reliance on studies conducted by Dr. Pavarina’s research group [28,29,30,31,32,33,34,35,37,40], which accounted for most of the included articles. The predominance of data generated using similar methodologies and by overlapping teams of investigators introduce an inherent risk of methodological and publication bias. This concentration of evidence from a single research network may limit the generalizability of the findings and could reduce the robustness and external validity of the conclusions drawn. Although these studies provide valuable and consistent data, the lack of broader independent validation underscores the need for future research from multiple centers and diverse clinical environments to confirm and expand upon these results. Therefore, the present review should be interpreted primarily as a call for further independent studies rather than as a basis for definitive clinical recommendations. Broader multicenter investigations are needed to validate and extend these findings before widespread clinical adoption can be advised.

### 4.4. Implications for Practice, Policy, and Future Research

The findings of this systematic review highlight significant implications for clinical practice, policy-making, and future research. Clinically, PDZ-aPDT presents a promising alternative or adjunct treatment for multidrug-resistant infections, biofilm-associated conditions, and superficial microbial infections such as oral candidiasis, denture stomatitis, and acne. Practitioners should consider integrating PDZ-aPDT into their treatment protocols while carefully selecting parameters based on available evidence. From a policy perspective, there is a critical need to fund and promote large-scale clinical trials to establish standardized guidelines and streamline regulatory approval processes for Photodithazine-based therapies, considering the urgency of antimicrobial resistance. Additionally, policies supporting specialized training in photodynamic therapy techniques could improve implementation efficacy. Future research should prioritize addressing gaps identified in this review through rigorous randomized controlled trials with larger patient populations, standardized treatment protocols, and investigations into optimal therapeutic parameters. Exploring the potential synergistic effects of PDZ-aPDT with other antimicrobials, DNase enzymes, and advanced delivery systems such as nanotechnology is also essential. Research aimed at overcoming current technical limitations, such as tissue penetration and efficacy in hypoxic environments, will further enhance the broader clinical applicability of PDZ-aPDT, thereby advancing patient care and public health outcomes.

## 5. Conclusions

This systematic review demonstrates that PDZ-aPDT is a promising therapeutic strategy with the potential for managing multidrug-resistant microbial infections, biofilm-associated conditions, and superficial infections including oral candidiasis, denture stomatitis, acne, and device-related infections. PDZ-aPDT exhibits broad-spectrum antimicrobial activity, favorable safety profiles, and minimal risk of inducing microbial resistance. Nevertheless, the existing body of evidence is limited by methodological heterogeneity, predominantly preclinical data, small sample sizes, and a lack of standardized treatment protocols. Thus, further high-quality clinical trials with rigorous methodology, larger patient populations, and long-term follow-up are essential to confirm efficacy, optimize treatment parameters, and ensure safety. Continued exploration into adjunctive and synergistic therapies, innovative delivery systems, and technological improvements for deeper tissue penetration and enhanced effectiveness under hypoxic conditions will further solidify the role of PDZ-aPDT as a valuable adjunct or alternative to conventional antimicrobials, effectively addressing the global challenge of antimicrobial resistance.

## Figures and Tables

**Table 1:** Search syntax used in the study.

Source	Search Term	Number of Results
PubMed	(“Photodynamic Therapy” [MeSH Terms] or “photodynamic therapy” [tiab] or “photodynamic inactivation” [tiab] or “light-based antimicrobial therapy” [tiab] or “aPDT” [tiab] or “photoactivated disinfection” [tiab] or “photo-inactivation” [tiab] or “photoantimicrobial” [tiab]) and (“Photodiathazine” [tiab] or photodithazine [tiab] or “PDZ” [tiab]) and (“Anti-Infective Agents” [MeSH Terms] or “microbial infection” [tiab] or “bacterial infection” [tiab] or “fungal infection” [tiab] or “biofilm” [tiab] or “Candida” [tiab] or “Staphylococcus” [tiab] or “Pseudomonas” [tiab] or “Escherichia coli” [tiab] or “antibacterial” [tiab] or “antifungal” [tiab] or “disinfection” [tiab] or “infection control” [tiab])	32
Embase	(‘photodynamic therapy’/exp or ‘photodynamic therapy’:ti,ab or ‘photodynamic inactivation’:ti,ab or ‘light-based antimicrobial therapy’:ti,ab or apdt:ti,ab or ‘photoactivated disinfection’:ti,ab or ‘photo-inactivation’:ti,ab or photoantimicrobial:ti,ab) and (‘photodiathazine’:ti,ab or photodithazine:ti,ab or pdz:ti,ab) and (‘antiinfective agent’/exp OR ‘microbial infection’:ti,ab or ‘bacterial infection’:ti,ab or ‘fungal infection’:ti,ab or biofilm:ti,ab or candida:ti,ab or staphylococcus:ti,ab or pseudomonas:ti,ab or ‘escherichia coli’:ti,ab or antibacterial:ti,ab or antifungal:ti,ab or disinfection:ti,ab or ‘infection control’:ti,ab)	46
Scopus	(TITLE-ABS-KEY(“photodynamic therapy” or “photodynamic inactivation” or “light-based antimicrobial therapy” or “aPDT” or “photoactivated disinfection” or “photo-inactivation” or “photoantimicrobial”)) and (TITLE-ABS-KEY(“photodiathazine” or “photodithazine” or “PDZ”)) and (TITLE-ABS-KEY(“anti-infective agents” or “microbial infection” or “bacterial infection” or “fungal infection” or “biofilm” or “Candida” or “Staphylococcus” or “Pseudomonas” or “Escherichia coli” or “antibacterial” or “antifungal” or “disinfection” OR “infection control”))	41
Cochrane Library	(“photodiathazine” or “photodithazine” or “PDZ”) and (“photodynamic therapy” or “PDT” or “photodynamic inactivation” or “photoactivated disinfection” or “photo-inactivation” or “photoantimicrobial” or “light-based antimicrobial therapy”) and (“microbial infection” or “bacterial infection” or “fungal infection” or “biofilm” or “Candida” or “Staphylococcus” or “Pseudomonas” or “Escherichia coli” or “antibacterial” or “antifungal” or “disinfection” or “infection control”)	8

**Table 2:** Evaluation of methodological quality and bias risk across included studies, with criteria breakdown.

Criteria	[28]	[29]	[30]	[31]	[32]	[33]	[34]	[35]	[36]	[37]	[38]	[39]	[40]
1.	1	1	1	1	1	1	1	1	1	1	1	1	1
2.	1	1	1	1	1	1	1	1	1	1	1	1	1
3.	1	1	1	1	1	1	1	1	1	1	1	1	1
4.	1	1	1	1	1	0	1	1	0	1	1	1	0
5.	1	1	1	1	1	1	1	1	1	1	1	1	1
6.	1	1	1	1	1	1	1	1	0	1	1	1	1
7.	0	1	1	1	1	1	1	1	0	0	1	1	1
8.	0	0	1	0	1	1	1	1	1	0	1	1	1
9.	0	0	0	0	0	0	0	0	0	1	1	1	1
Total Score	6	7	8	7	8	7	8	8	5	7	9	9	8
Risk of Bias	Moderate	Low	Low	Low	Low	Low	Low	Low	Moderate	Low	Low	Low	Low

**Table 3:** A general overview of the included research.

Study	Country	Type	Site	Aims
Abreu-Pereira et al., 2025 [28]	Brazil	In vitro	-	To evaluate the effect of ZER combined with PDZ-aPDT on biofilms formed by fluconazole-resistant and -susceptible *Candida albicans*, including clinical isolates.
Alves et al., 2020 [29]	Brazil	RCT	-	To evaluate the efficacy of PDZ-aPDT as a treatment for denture stomatitis in patients, compared to conventional topical nystatin therapy, through a randomized clinical trial.
Carmello et al., 2016 [30]	Brazil	In vivo	Tongue	To evaluate the effectiveness of PDZ-aPDT in treating oral candidiasis in an immunosuppressed mouse model by comparing it with nystatin treatment and assessing its effects on fungal viability, lesion remission, tissue response, and inflammatory cytokine expression.
Dias et al., 2020 [31]	Brazil	In vitro	-	To evaluate whether successive applications of PDZ-aPDT affect the susceptibility, resistance, or tolerance of *Candida albicans* in planktonic cultures and biofilms, and to assess the influence of fluconazole on fungal recovery after treatment.
Dias et al., 2023 [32]	Brazil	In vitro	-	To evaluate the effect of ten consecutive applications of sub-lethal aPDT, mediated by PDZ and CUR, on the viability, ROS production, and gene expression of *Candida albicans* in both planktonic cultures and biofilms.
Jordão et al., 2020 [33]	Brazil	In vitro	-	To evaluate whether PDZ-aPDT or CUR affects the expression of *Candida albicans* genes related to adhesion, biofilm formation, and oxidative stress responses in biofilms.
Jordão et al., 2023 [34]	Brazil	In vivo	Tongue	To evaluate whether the application of DNase I enzyme could enhance the efficacy of PDZ-aPDT in treating oral candidiasis in mice infected with fluconazole-susceptible and -resistant *Candida albicans* strains.
Jordão et al., 2024 [35]	Brazil	In vivo	Tongue	To evaluate the effects of combining DNase I with aPDT on *Candida albicans* gene expression and the local inflammatory response in mice with oral candidiasis.
Lee et al., 2024 [36]	Korea	In vivo	Acne lesions	To evaluate the effectiveness of aPDT using a topical Photodithazine gel and micro-LED irradiation in reducing inflammation caused by acne bacteria (*Cutibacterium acnes*) in a mouse model.
Quishida et al., 2015 [37]	Brazil	In vitro	-	To evaluate the effectiveness of one and three successive applications of PDZ-aPDT and LED light against a multispecies biofilm of *Candida albicans*, *Candida glabrata*, and *Streptococcus mutans* formed on denture base acrylic resin.
Souza et al., 2021 [38]	Brazil	In vitro	-	To analyze, in vitro, the effect of PDZ-aPDT on the viability, metabolism, and ROS production of MSSA and MRSA strains.
Souza et al., 2024 [39]	Brazil	In vitro and in vivo	Larvae	To analyze, both in vitro and in vivo, the effects of PDZ-aPDT on MRSA, evaluating its impact on biofilms and in an experimental infection model using *Galleria mellonella* larvae.
Tavaresa et al., 2018 [40]	Brazil	In vitro	-	To evaluate the effects of PDZ-aPDT, alone or in combination with metronidazole administration, against biofilms of *Fusobacterium nucleatum* and *Porphyromonas gingivalis.*

aPDT—antimicrobial photodynamic therapy, RCT- randomized clinical trial, API—Antimicrobial Photodynamic Inactivation, PDZ—Photodithazine^®^, CUR—Curcumin, ROS—reactive oxygen species, MSSA—Methicillin-Sensitive Staphylococcus Aureus, MRSA—Methicillin-Resistant Staphylococcus Aureus, and PDZ-aPDT—antimicrobial photodynamic therapy with Photodithazine^®^.

**Table 4:** Summary of principal results and study details.

Study	Study Groups	Main Outcomes
Abreu-Pereira et al., 2025 [28]	Control, ZER, PDZ, LED, ZER + aPDT, ZER + PDZ, ZER + LED, and aPDT	ZER + PDZ-aPDT reduced biofilm viability and biomass in both fluconazole-susceptible and -resistant *Candida albicans* strains. ZER + aPDT achieved greater reductions in colony counts (2.01 log10), total/insoluble biomass, and key biofilm components (proteins, polysaccharides, and eDNA) compared to single treatments. Confocal microscopy confirmed a higher proportion of dead cells in biofilms treated with ZER + aPDT. ZER enhances aPDT effectiveness by disrupting the biofilm’s extracellular matrix, suggesting a promising strategy against resistant *Candida* biofilms.
Alves et al., 2020 [29]	aPDT, NYS	PDZ-aPDT was more effective than nystatin in reducing the total microbiota on both palates and dentures of patients with denture stomatitis. Both aPDT and nystatin were equally effective in reducing *Candida* species on the palate and denture, with similar clinical improvement rates (about 53–54%). Recurrence of denture stomatitis was observed in both treatment groups during follow-up, indicating a need for improved or additional protocols. aPDT using Photodithazine was safe and well-tolerated, showing promise as an alternative or adjunctive therapy for denture stomatitis.
Carmello et al., 2016 [30]	aPDT (P+L+), NYS, control (P-L-), light only (P-L+), PDZ only (P+L-), and NC	PDZ-aPDT was as effective as nystatin in reducing *Candida albicans* in oral candidiasis in mice, with both treatments achieving about a 3 log10 reduction in fungal viability 24 h after therapy. aPDT led to complete remission of oral lesions in all treated mice, while nystatin resulted in only partial lesion remission. Histological analysis showed mild inflammation and no tissue damage in aPDT-treated tongues, suggesting the treatment is safe for host tissues. aPDT promoted beneficial TNF-α expression and lower levels of pro-inflammatory cytokines compared to the control, indicating a positive immunomodulatory effect.
Dias et al., 2020 [31]	P+L+ (photosensitizer + light), P+L- (photosensitizer only), P-L+ (light only), and P-L- (no treatment), with and without fluconazole	· Successive PDZ-aPDT effectively inactivated *Candida albicans.* Complete inactivation was achieved after 3 cycles for planktonic cells and 5 cycles for biofilms. Biofilms were more tolerant to aPDT than planktonic cells, requiring more treatment cycles for full inactivation. Combining aPDT with fluconazole further enhanced antifungal efficacy, showing greater log reductions in fungal viability compared to aPDT alone. No development of resistance or tolerance to aPDT was observed in *C. albicans* after repeated treatments; susceptibility increased, especially with the antifungal present.
Dias et al., 2023 [32]	P+L+ (PDZ + red LED), C+L+ (CUR + blue LED), P+L- (PDZ only), C+L- (CUR only), P-L+ (red LED only), C-L+ (blue LED only), P-L- (no treatment), and C-L- (no treatment)	PDZ-aPDT was highly effective in reducing the viability of *Candida albicans* biofilms, leading to the complete absence of viable cells after seven applications, while CUR-mediated aPDT led to increased biofilm viability after repeated treatments, suggesting possible resistance induction. Higher intracellular ROS production was observed in biofilms treated with PDZ-aPDT compared to planktonic cells, indicating stronger oxidative stress in biofilm conditions. Gene expression analysis showed that PDZ-aPDT increased SOD1 expression and reduced CAP1 and ERG11 expression in biofilms, regardless of fluconazole presence, indicating an oxidative stress response and potential impact on antifungal resistance mechanisms. Sub-lethal doses of CUR-aPDT could induce resistance in *C. albicans* biofilms, while PDZ-aPDT remained effective, highlighting the importance of the choice of photosensitizer for controlling fungal biofilms.
Jordão et al., 2020 [33]	P+L+ (PDZ + LED), P+L- (PDZ only), P-L+ (LED only), P-L- (no treatment), P+L+ (CUR + LED), P+L− (CUR only), P-L+ (LED only), and P−L− (no treatment) (each with varying concentrations and fluences)	PDZ-aPDT reduced the expression of key *Candida albicans* virulence genes (ALS1 and HWP1 for adhesion/biofilm and CAP1, CAT1, and SOD1 for oxidative stress response) in biofilms, especially at higher concentrations (200 mg/L) combined with LED light. Both PDZ and CUR as photosensitizers with LED light led to downregulation of all evaluated genes, indicating a strong reduction in virulence and adaptability to oxidative stress in *C. albicans* biofilms. Reduction in gene expression was most pronounced with higher photosensitizer concentrations and higher LED fluence, but even lower concentrations with higher light intensity were effective. The study supports that PDZ-aPDT can reduce the virulence and oxidative stress adaptability of *C. albicans*, suggesting potential as an alternative or adjunctive treatment for fungal biofilm-related infections.
Jordão et al., 2023 [34]	DNase+P+L+ (DNase + PDZ-aPDT), P+L+ (PDZ-aPDT), P+L− (PDZ only), P−L+ (LED only), DNase (DNase only), P−L− (no treatment), NIC+ (healthy, immunosuppressed), and NIC− (healthy, not immunosuppressed)	Combining DNase I enzyme with PDZ-aPDT enhanced treatment efficacy against both fluconazole-susceptible and -resistant *Candida albicans* in a mouse model of oral candidiasis, with immediate fungal viability reductions of 4.26 log10 (susceptible) and 2.89 log10 (resistant). The combination therapy led to near-complete remission of oral lesions and mild inflammation in both groups, outperforming PDZ-aPDT alone, which achieved only partial lesion remission. Microscopy showed that DNase + PDZ-aPDT reduced the presence of fungal polysaccharides and cells in tongue tissue to levels like healthy controls, suggesting effective biofilm and ECM disruption. Seven days after treatment, the combined therapy group maintained a lower fungal load and less lesion recurrence than groups treated with PDZ-aPDT alone or DNase alone, highlighting DNase as a promising adjuvant for improving aPDT outcomes in resistant fungal infections.
Jordão et al., 2024 [35]	NIC (healthy control), P−L− (infected, untreated), P+L+ (PDZ-aPDT), P−L+ (LED only), P+L− (PDZ only), DNase (DNase only), and DNase+P+L+ (DNase + PDZ-aPDT)	Combining DNase I with PDZ-aPDT reduced the expression of *Candida albicans* virulence genes (including genes related to adhesion, biofilm matrix production, and oxidative stress response) in both fluconazole-susceptible and -resistant strains, immediately and 7 days post-treatment. The combination therapy increased the local production of inflammatory cytokines (IL-6, TNF-α, and MCP-1) in mouse oral tissue, particularly in infections with susceptible strains, suggesting an enhanced immunomodulatory and host defense response. Reductions in gene expression and increased cytokine production correlated with improved infection control and decreased recurrence of oral lesions in the treated mice. This approach demonstrated that DNase I enhances the efficacy of aPDT, offering a promising strategy to improve outcomes against both drug-susceptible and drug-resistant *C. albicans* infections by targeting fungal virulence and boosting host immunity.
Lee et al., 2024 [36]	Normal control, C. acnes only, FDT gel only, LED 10 min, LED 15 min, aPDT 10 min (FDT gel + LED), and aPDT 15 min (FDT gel + LED)	PDZ-aPDT with micro-LED reduced the size and number of acne lesions in a mouse model compared to controls treated with LED or photosensitizer alone. Inflammatory biomarkers (IL-1α, IL-1β, IL-8, TNF-α, TLR2, and MMP-2) showed marked decreases in both mRNA and protein levels after aPDT, with the most reduction after 15 min of treatment. Histological and immunohistochemical analyses revealed a pronounced reduction in inflammatory cell infiltration and expression of key inflammatory markers (NLRP3, caspase-1, SREBP1, IL-1α, and TNF-α) in skin tissue following aPDT, especially with longer LED exposure. PDZ-aPDT demonstrated a shorter required incubation time and strong anti-inflammatory effects, supporting its potential as a rapid and effective treatment for acne-induced inflammation.
Quishida et al., 2015 [37]	P+L+ (PDZ + light), P+L− (PDZ only), P−L+ (light only), and P−L− (untreated control), each with one or three applications	Three consecutive applications of PDZ-aPDT with LED light reduced the viability and total biomass of multispecies biofilms (composed of *Candida albicans*, *Candida glabrata*, and *Streptococcus mutans*) formed on acrylic resin compared to a single application. Metabolic activity of the biofilms was reduced after both one and three applications, but the reduction was greater after three applications. Confocal laser scanning microscopy revealed a visual increase in dead cells within the biofilm after PDZ-aPDT, especially after three applications, supporting the quantitative findings. The study suggests that multiple sessions of PDZ-aPDT are more effective for biofilm control on denture materials, though further protocol improvements are needed before clinical use.
Souza et al., 2021 [38]	aPDT (PDZ + light), PDZ only, light only, and untreated control, each with MSSA and MRSA, and varying PDZ concentrations and light fluences	PDZ-aPDT was efficiently internalized by both MSSA and MRSA at all tested concentrations, with 15 min of incubation sufficient for uptake. PDZ-aPDT resulted in complete bacterial inactivation (no viable colonies) of both MSSA and MRSA in most tested conditions, except at the lowest fluences and concentrations, showing similar effectiveness regardless of the antibiotic resistance profile. Reductions in bacterial metabolic activity and increased production of ROS were observed after aPDT, with the magnitude of ROS production and bacterial inactivation being dependent on both light fluence and PDZ concentration. PDZ showed no cytotoxic effect in the absence of light, supporting its safety profile, and the study recommends specific combinations (≥50 mg/L PDZ and ≥25–50 J/cm^2^ light) for potential clinical applications to treat superficial *S. aureus* infections.
Souza et al., 2024 [39]	aPDT (PDZ + light), PDZ only, light only, untreated control (PBS), and MRSA only (infection), each with varying PDZ concentrations and light fluences, both in vitro and in vivo (*Galleria mellonella*)	PDZ-aPDT caused a reduction in MRSA biofilm viability, up to 6.7 log10, with decreased metabolic activity and increased ROS production but had minimal effect on total biofilm biomass. Confocal and electron microscopy showed PDZ effectively penetrated biofilms, and aPDT produced a qualitative disruption of biofilm structure despite persistent biomass, as measured by crystal violet staining. In a Galleria mellonella in vivo model, optimized (lower) doses of PDZ and light improved larval survival (up to 40% at 7 days), increased immune cell (hemocyte) counts, and enhanced larval health compared to infected controls. The study demonstrates that PDZ-aPDT is effective against MRSA biofilms in vitro and improves infection outcomes in vivo by both direct bacterial inactivation and stimulation of the host immune response, supporting its potential as an alternative to antibiotics for drug-resistant infections.
Tavaresa et al., 2018 [40]	aPDT (PDZ + light), PDZ only, light only, untreated control, MTZ only, and aPDT + MTZ (combination therapy), each at varying PDZ and MTZ concentrations, for both F. nucleatum and P. gingivalis biofilms	PDZ-aPDT alone caused modest reductions in the viability of mature biofilms, 1.12 log10 for *F. nucleatum* and 2.66 log10 for *P. gingivalis,* with the greatest effect at 100 mg/L PDZ and red LED light. Combining PDZ-aPDT with high-dose metronidazole (MTZ) greatly enhanced the antimicrobial effect, achieving up to 3.94 log10 reduction for *F. nucleatum* and up to 5 log10 reduction for *P. gingivalis* biofilms. Neither aPDT nor MTZ alone was able to fully disrupt biofilm structure, but their combination led to bactericidal activity, confirmed by live/dead staining and confocal microscopy. Localized antibiotic administration can act as an effective adjuvant to aPDT for controlling resistant anaerobic oral biofilms, highlighting the value of combination therapies for periodontal and peri-implant infections.

aPDT—antimicrobial photodynamic therapy, PDZ—Photodithazine^®^, ZER—Zerumbone, LED—Light Emitting Diode, NYS—nystatin, NC—Negative Control, CUR—Curcumin, C+L+—CUR + LED, C+L−—CUR only, C−L+—LED only, C−L−—no treatment (CUR group), P+L+—PDZ + LED (or light), P+L−—PDZ only, P−L+—light only, P−L−—no treatment (PDZ group), NIC+—healthy, immunosuppressed control, NIC−—healthy, non-immunosuppressed control, FDT—topical Photodithazine gel, MSSA—Methicillin-sensitive Staphylococcus Aureus, MRSA—Methicillin-resistant Staphylococcus Aureus, and MTZ—metronidazole.

**Table 5:** aPDT characteristics.

Study	Light Source	Wavelength [nm]	Energy Fluence [J/cm^2^]	PS Dose/Concentration and Administration	Incubation Time
Abreu-Pereira et al., 2025 [28]	LED	600	50	PDZ 200 mg/L, after ZER pretreatment	20 min (ZER), then PDZ
Alves et al., 2020 [29]	LED	660	50	PDZ 200 mg/L, topical on palate/denture	20 min
Carmello et al., 2016 [30]	LED	660	50	PDZ 200 mg/L, topical	20 min
Dias et al., 2020 [31]	LED	660	34	PDZ 25 mg/L, planktonic/biofilm	20 min (pre-irr.), exposure time not specified
Dias et al., 2023 [32]	Red LED	660	18	PDZ 25 mg/L, in PBS	20 min (pre-irr.), 9 min LED
Jordão et al., 2020 [33]	LED	660	37.5, 50	PDZ 100 or 200 mg/L, applied to biofilms	20 min (standard for group; check methods for confirmation)
Jordão et al., 2023 [34]	LED	660	50	PDZ 200 mg/L, applied after DNase	20 min (PDZ), 5 min (DNase)
Jordão et al., 2024 [35]	LED	660	50	PDZ 200 mg/L, applied after DNase	20 min (PDZ), 5 min (DNase)
Lee et al., 2024 [36]	Micro-LED	650	Not stated	PDZ, topical gel (conc. not stated)	Not stated
Quishida et al., 2015 [37]	LED	660	37.5	PDZ 175 or 200 mg/L, applied to biofilms	20 min
Souza et al., 2021 [38]	Not stated	Not stated	25, 50, and 100	PDZ: 25, 50, 75, and 100 mg/L; planktonic MSSA/MRSA strains	15 min
Souza et al., 2024 [39]	Biotable Biopdi660 (LED array)	660	25, 50, and 100 (in vitro); 10 (in vivo)	PDZ: 50, 75 μg/mL (in vitro biofilm); 5, 0.25, and 2.5 × 10^−7^ μg/mL (in vivo)	15 min (in vitro and in vivo)
Tavaresa et al., 2018 [40]	LED	660	50	PDZ: 50, 75, and 100 mg/L; 5-day biofilm (F. nucleatum, P. gingivalis)	10 min (in darkness)

LED—Light Emitting Diode, PDZ—Photodithazine^®^, ZER—Zerumbone, PBS—Phosphate-Buffered Saline, and DNase—Deoxyribonuclease.

**Table 6:** Properties of Photodithazine as photosensitizer.

Property	Description/Details
Chemical Structure	Chlorin e6 derivative (second-generation photosensitizer)
Solubility	Water-soluble
Activation Wavelength	Red light, typically 660 nm
Mechanism of Action	Upon irradiation produces singlet oxygen and reactive oxygen species (ROS) that damage cell walls
Spectrum of Activity	Effective against Gram-positive and Gram-negative bacteria, fungi (*Candida* spp.), and biofilms
Formulation	Used as solution or topical gel
Typical Concentration	10–100 mg/L for in vitro studies; clinical gels up to 1%
Application/Incubation	Applied for 10–20 min before irradiation (pre-irradiation phase)
Light Dose Used	Typically 50 J/cm^2^
Advantages	High antimicrobial efficacy, low dark cytotoxicity, acts on drug-resistant microbes, and biofilm action
Brand Name	Photodithazine^®^
Safety	Low toxicity in the absence of light

Source: [28,29,30,31,32,33,34,35,36,37,38,39,40], ROS—reactive oxygen species.

## References

[1] Salam M.A., Al-Amin M.Y., Salam M.T., Pawar J.S., Akhter N., Rabaan A.A., Alqumber M.A.A. (2023). Antimicrobial Resistance, A Growing Serious Threat for Global Public Health. Healthcare.

[2] Coque T.M., Cantón R., Pérez-Cobas A.E., Fernández-de-Bobadilla M.D., Baquero F. (2023). Antimicrobial Resistance in the Global Health Network, Known Unknowns and Challenges for Efficient Responses in the 21st Century. Microorganisms.

[3] Muteeb G., Rehman M.T., Shahwan M., Aatif M. (2023). Origin of Antibiotics and Antibiotic Resistance, and Their Impacts on Drug Development, A Narrative Review. Pharmaceuticals.

[4] Oliveira M., Antunes W., Mota S., Madureira-Carvalho Á., Dinis-Oliveira R.J., Dias da Silva D. (2024). An Overview of the Recent Advances in Antimicrobial Resistance. Microorganisms.

[5] Donlan R.M., Costerton J.W. (2002). Biofilms, Survival Mechanisms of Clinically Relevant Microorganisms. Clin. Microbiol. Rev..

[6] Gulati M., Nobile C.J. (2016). Candida albicans Biofilms, Development, Regulation, and Molecular Mechanisms. Microbes Infect..

[7] Drago L., Fidanza A., Giannetti A., Ciuffoletti A., Logroscino G., Romanò C.L. (2024). Correction: Drago et al. Bacteria Living in Biofilms in Fluids, Could Chemical Antibiofilm Pretreatment of Culture Represent a Paradigm Shift in Diagnostics?. Microorganisms.

[8] Liu Y., Qin R., Zaat S.A.J., Breukink E., Heger M. (2015). Antibacterial Photodynamic Therapy, Overview of a Promising Approach to Fight Antibiotic-Resistant Bacterial Infections. J. Clin. Transl. Res..

[9] Youf R., Müller M., Balasini A., Thétiot F., Müller M., Hascoët A., Jonas U., Schönherr H., Lemercier G., Montier T. (2021). Antimicrobial Photodynamic Therapy, Latest Developments with a Focus on Combinatory Strategies. Pharmaceutics.

[10] Afrasiabi S., Partoazar A., Chiniforush N., Goudarzi R. (2022). The Potential Application of Natural Photosensitizers Used in Antimicrobial Photodynamic Therapy Against Oral Infections. Pharmaceuticals.

[11] Kashef N., Hamblin M.R. (2017). Can Microbial Cells Develop Resistance to Oxidative Stress in Antimicrobial Photodynamic Inactivation?. Drug Resist. Updates.

[12] Silva L.B.B.D., Castilho I.G., Souza Silva F.A.D., Ghannoum M., Garcia M.T., Carmo P.H.F.D. (2025). Antimicrobial Photodynamic Therapy for Superficial, Skin, and Mucosal Fungal Infections, An Update. Microorganisms.

[13] Michalak M., Szymczyk J., Pawska A., Wysocki M., Janiak D., Ziental D., Ptaszek M., Güzel E., Sobotta L. (2025). Chlorin Activity Enhancers for Photodynamic Therapy. Molecules.

[14] Pires L., Bosco Sde M., da Silva N.F., Kurachi C. (2013). Photodynamic Therapy for Pythiosis. Vet. Dermatol..

[15] Fontana L.C., Pinto J.G., Pereira A.H.C., Soares C.P., Raniero L.J., Ferreira-Strixino J. (2017). Photodithazine Photodynamic Effect on Viability of 9L/lacZ Gliosarcoma Cell Line. Lasers Med. Sci..

[16] Fiegler-Rudol J., Kapłon K., Kotucha K., Moś M., Skaba D., Kawczyk-Krupka A., Wiench R. (2025). Hypocrellin-Mediated PDT: A Systematic Review of Its Efficacy, Applications, and Outcomes. Int. J. Mol. Sci..

[17] Turubanova V.D., Balalaeva I.V., Mishchenko T.A., Catanzaro E., Alzeibak R., Peskova N.N., Efimova I., Bachert C., Mitroshina E.V., Krysko O. (2019). Immunogenic Cell Death Induced by a New Photodynamic Therapy Based on Photosens and Photodithazine. J. Immunother. Cancer.

[18] Santos Vitorio G.D., de Almeida R.M.S., Pinto J.G., Fontana L.C., Ferreira-Strixino J. (2021). Analysis of the Effects of Photodynamic Therapy with Photodithazine on the Treatment of 9l/lacZ Cells, In Vitro Study. Photodiagn. Photodyn. Ther..

[19] Szewczyk G., Mokrzyński K. (2025). Concentration-Dependent Photoproduction of Singlet Oxygen by Common Photosensitizers. Molecules.

[20] Buzzá H.H., Silva L.V., Moriyama L.T., Bagnato V.S., Kurachi C. (2014). Evaluation of Vascular Effect of Photodynamic Therapy in Chorioallantoic Membrane Using Different Photosensitizers. J. Photochem. Photobiol. B.

[21] Fiegler-Rudol J., Skaba D., Wiench R. (2025). Antimicrobial Efficacy of Nd:YAG Laser in Polymicrobial Root Canal Infections: A Systematic Review of In Vitro Studies. Int. J. Mol. Sci..

[22] Abreu-Pereira C.A., Gorayb-Pereira A.L., Jordão C.C., Bitencourt G.P., Cilli E.M., Pavarina A.C. (2025). Efficiency of the Extracellular Polymeric Matrix Disruptor Zerumbone in Combination with Photodithazine^®^ in the Photodynamic Inactivation of Monospecies Biofilms. Lasers Med. Sci..

[23] Schardt C., Adams M.B., Owens T., Keitz S., Fontelo P. (2007). Utilization of the PICO Framework to Improve Searching PubMed for Clinical Questions. BMC Med. Inform. Decis. Mak..

[24] Page M.J., Shamseer L., Tricco A.C. (2018). Registration of Systematic Reviews in PROSPERO, 30,000 Records and Counting. Syst. Rev..

[25] Page M.J., McKenzie J.E., Bossuyt P.M., Boutron I., Hoffmann T.C., Mulrow C.D., Shamseer L., Tetzlaff J.M., Akl E.A., Brennan S.E. (2021). The PRISMA 2020 Statement, An Updated Guideline for Reporting Systematic Reviews. BMJ.

[26] Watson P.F., Petrie A. (2010). Method Agreement Analysis, A Review of Correct Methodology. Theriogenology.

[27] Higgins J., Thomas J., Chandler J., Cumpston M., Li T., Page M. (2023). Welch Cochrane Handbook for Systematic Reviews of Interventions Version 6.4. Cochrane. www.training.cochrane.org/handbook.

[28] Abreu-Pereira C.A., Gorayb-Pereira A.L., Jordão C.C., Paro C.B., Barbugli P.A., Pavarina A.C. (2025). Zerumbone Enhances the Photodynamic Effect Against Biofilms of Fluconazole-Resistant Candida albicans Clinical Isolates. J. Dent..

[29] Alves F., Carmello J.C., Alonso G.C., Mima E.G.O., Bagnato V.S., Pavarina A.C. (2020). A Randomized Clinical Trial Evaluating Photodithazine-Mediated Antimicrobial Photodynamic Therapy as a Treatment for Denture Stomatitis. Photodiagn. Photodyn. Ther..

[30] Carmello J.C., Alves F., GBasso F., de Souza Costa C.A., Bagnato V.S., de Oliveira Mima E.G., Pavarina A.C. (2016). Treatment of Oral Candidiasis Using Photodithazine^®^-Mediated Photodynamic Therapy In Vivo. PLoS ONE.

[31] Dias L.M., Klein M.I., Jordão C.C., Carmello J.C., Bellini A., Pavarina A.C. (2020). Successive Applications of Antimicrobial Photodynamic Therapy Effects the Susceptibility of Candida albicans Grown in Medium with or Without Fluconazole. Photodiagn. Photodyn. Ther..

[32] Dias L.M., Klein M.I., Ferrisse T.M., Medeiros K.S., Jordão C.C., Bellini A., Pavarina A.C. (2023). The Effect of Sub-Lethal Successive Applications of Photodynamic Therapy on Candida albicans Biofilm Depends on the Photosensitizer. J. Fungi.

[33] Jordão C.C., de Sousa T.V., Klein M.I., Dias L.M., Pavarina A.C., Carmello J.C. (2020). Antimicrobial Photodynamic Therapy Reduces Gene Expression of Candida albicans in Biofilms. Photodiagn. Photodyn. Ther..

[34] Jordão C.C., Klein M.I., Barbugli P.A., Mima E.G.D.O., de Sousa T.V., Ferrisse T.M., Pavarina A.C. (2023). DNase Improves the Efficacy of Antimicrobial Photodynamic Therapy in the Treatment of Candidiasis Induced with Candida albicans. Front. Microbiol..

[35] Jordão C.C., Klein M.I., Barbugli P.A., Ferrisse T.M., de Moraes J.C.G., Pavarina A.C. (2025). The Association of DNase I with Antimicrobial Photodynamic Therapy Affects Candida albicans Gene Expression and Promotes Immunomodulatory Effects in Mice with Candidiasis. Photochem. Photobiol. Sci..

[36] Lee S.M., Kim S.-H., Kim Z., Lee J.-B. (2024). Photodynamic Effects of Topical Photosensitizer, Photodithazine Using Micro-LED for Acne Bacteria Induced Inflammation. Ann. Dermatol..

[37] Quishida C.C.C., Mima E.G.O., Dovigo L.N., Jorge J.H., Bagnato V.S., Pavarina A.C. (2015). Photodynamic Inactivation of a Multispecies Biofilm Using Photodithazine^®^ and LED Light After One and Three Successive Applications. Lasers Med. Sci..

[38] Souza B.M.N., Pinto J.G., Pereira A.H.C., Miñán A.G., Ferreira-Strixino J. (2021). Efficiency of Antimicrobial Photodynamic Therapy with Photodithazine^®^ on MSSA and MRSA Strains. Antibiotics.

[39] Souza B.M.N., Miñán A.G., Brambilla I.R., Pinto J.G., Garcia M.T., Junqueira J.C., Ferreira-Strixino J. (2024). Effects of Antimicrobial Photodynamic Therapy with Photodithazine^®^ on Methicillin-Resistant Staphylococcus aureus (MRSA), Studies in Biofilms and Experimental Model with Galleria mellonella. J. Photochem. Photobiol. B.

[40] Tavares L.J., de Avila E.D., Klein M.I., Panariello B.H.D., Spolidorio D.M.P., Pavarina A.C. (2018). Antimicrobial Photodynamic Therapy Alone or in Combination with Antibiotic Local Administration Against Biofilms of Fusobacterium nucleatum and Porphyromonas gingivalis. J. Photochem. Photobiol. B.

[41] Alves F., Alonso G.C., Carmello J.C., Mima E.G.O., Bagnato V.S., Pavarina A.C. (2018). Antimicrobial Photodynamic Therapy Mediated by Photodithazine^®^ in the Treatment of Denture Stomatitis, A Case Report. Photodiagn. Photodyn. Ther..

[42] Zhang H., Zhao Y., Tao H., Feng C., Wang P., Zhang L., Liu X., Chen Y., Wang X. (2024). A Chlorin e6 Derivative-Mediated Photodynamic Therapy for Mild to Moderate Acne, A Prospective, Single-Blind, Randomized, Split-Face Controlled Study. Photodiagn. Photodyn. Ther..

[43] Potapov A., Matveev L., Moiseev A., Sedova E., Loginova M., Karabut M., Kuznetsova I., Levchenko V., Grebenkina E., Gamayunov S. (2023). Multimodal OCT Control for Early Histological Signs of Vulvar Lichen Sclerosus Recurrence After Systemic PDT, Pilot Study. Int. J. Mol. Sci..

[44] Ferreira J., Menezesa P.F.C., Sibatac C.H., Allison R.R., Zucoloto S., Castro e Silva O., Bagnato V.S. (2009). Can Efficiency of the Photosensitizer Be Predicted by Its Photostability in Solution?. Laser Phys..

[45] Łopaciński M., Fiegler-Rudol J., Niemczyk W., Skaba D., Wiench R. (2025). Riboflavin- and Hypericin-Mediated Antimicrobial Photodynamic Therapy as Alternative Treatments for Oral Candidiasis, A Systematic Review. Pharmaceutics.

[46] Wiench R., Nowicka J., Pajączkowska M., Kuropka P., Skaba D., Kruczek-Kazibudzka A., Kuśka-Kiełbratowska A., Grzech-Leśniak K. (2021). Influence of Incubation Time on Ortho-Toluidine Blue Mediated Antimicrobial Photodynamic Therapy Directed Against Selected Candida Strains—An In Vitro Study. Int. J. Mol. Sci..

[47] Kubizna M., Dawiec G., Wiench R. (2024). Efficacy of Curcumin-Mediated Antimicrobial Photodynamic Therapy on Candida spp., A Systematic Review. Int. J. Mol. Sci..

[48] Fiegler-Rudol J., Grzech-Leśniak Z., Tkaczyk M., Grzech-Leśniak K., Zawilska A., Wiench R. (2025). Enhancing Root Canal Disinfection with Er:YAG Laser, A Systematic Review. Dent. J..

[49] Dembicka-Mączka D., Kępa M., Fiegler-Rudol J., Grzech-Leśniak Z., Matys J., Grzech-Leśniak K., Wiench R. (2025). Evaluation of the Disinfection Efficacy of Er: YAG Laser Light on Single-Species Candida Biofilms—An In Vitro Study. Dent. J..

[50] Quishida C.C., Carmello J.C., Mima E.G., Bagnato V.S., Machado A.L., Pavarina A.C. (2015). Susceptibility of Multispecies Biofilm to Photodynamic Therapy Using Photodithazine^®^. Lasers Med. Sci..

[51] Huang L., Xuan Y., Koide Y., Zhiyentayev T., Tanaka M., Hamblin M.R. (2012). Type I and Type II Mechanisms of Antimicrobial Photodynamic Therapy, An In Vitro Study on Gram-Negative and Gram-Positive Bacteria. Lasers Surg. Med..

[52] Dougherty T.J., Gomer C.J., Henderson B.W., Jori G., Kessel D., Korbelik M., Moan J., Peng Q. (1998). Photodynamic Therapy. J. Natl. Cancer Inst..

[53] Zhang J., Zhang Y., Zhang H., Zhai W., Shi X., Li C. (2023). A Hypoxia-Activatable Theranostic Agent with Intrinsic Endoplasmic Reticulum Affinity and Type-I Photosensitivity. J. Mater. Chem. B.

[54] Mima E.G., Vergani C.E., Machado A.L., Massucato E.M., Colombo A.L., Bagnato V.S., Pavarina A.C. (2012). Comparison of Photodynamic Therapy Versus Conventional Antifungal Therapy for the Treatment of Denture Stomatitis, A Randomized Clinical Trial. Clin. Microbiol. Infect..

[55] Rai A., Misra S.R., Panda S., Sokolowski G., Mishra L., Das R., Lapinska B. (2022). Nystatin Effectiveness in Oral Candidiasis Treatment, A Systematic Review & Meta-Analysis of Clinical Trials. Life.

[56] Dovigo L.N., Carmello J.C., de Souza Costa C.A., Vergani C.E., Brunetti I.L., Bagnato V.S., Pavarina A.C. (2013). Curcumin-Mediated Photodynamic Inactivation of Candida albicans in a Murine Model of Oral Candidiasis. Med. Mycol..

[57] Agostinis P., Berg K., Cengel K.A., Foster T.H., Girotti A.W., Gollnick S.O., Hahn S.M., Hamblin M.R., Juzeniene A., Kessel D. (2011). Photodynamic Therapy of Cancer, An Update. CA Cancer J. Clin..

[58] Hong L., Li J., Luo Y., Guo T., Zhang C., Ou S., Long Y., Hu Z. (2022). Recent Advances in Strategies for Addressing Hypoxia in Tumor Photodynamic Therapy. Biomolecules.

[59] Mallidi S., Anbil S., Bulin A.L., Obaid G., Ichikawa M., Hasan T. (2016). Beyond the Barriers of Light Penetration, Strategies, Perspectives and Possibilities for Photodynamic Therapy. Theranostics.

[60] Hamblin M.R. (2020). Photodynamic Therapy for Cancer, What’s Past is Prologue. Photochem. Photobiol..

[61] Gubarkova E.V., Feldchtein F.I., Zagaynova E.V., Gamayunov S.V., Sirotkina M.A., Sedova E.S., Kuznetsov S.S., Moiseev A.A., Matveev L.A., Zaitsev V.Y. (2019). Optical Coherence Angiography for Pre-Treatment Assessment and Treatment Monitoring Following Photodynamic Therapy, A Basal Cell Carcinoma Patient Study. Sci. Rep..

[62] Zhang Y., Zheng J., Jin F., Xiao J., Lan N., Xu Z., Yue X., Li Z., Li C., Cao D. (2024). Fiber-Optic Drug Delivery Strategy for Synergistic Cancer Photothermal-Chemotherapy. Light Sci. Appl..

[63] Overchuk M., Weersink R.A., Wilson B.C., Zheng G. (2023). Photodynamic and Photothermal Therapies, Synergy Opportunities for Nanomedicine. ACS Nano.

[64] Wiench R., Kuśka-Kiełbratowska A., Kępa M., Grzech-Leśniak Z., Jabłoński M., Kiryk J., Grzech-Leśniak K., Skaba D. (2025). Comparison of the Efficacy of Simple and Combined Oral Rinses with Chlorhexidine Digluconate Against Selected Bacterial and Yeast Species, An In Vitro Study. Dent. Med. Probl..

